# Supplementary material for: HUWE1 E3 ligase promotes PINK1/PARKIN-independent mitophagy by regulating AMBRA1 activation via IKKα
Source: Nat Commun. 2018 Sep 14;9:3755. doi: 10.1038/s41467-018-05722-3 (PMC6138665; doi:10.1038/s41467-018-05722-3)
Supplement: Supplementary file 1 — Supplementary Information [file 41467_2018_5722_MOESM1_ESM.pdf]

Supplementary Information

HUWE1 E3 ligase promotes PINK1/PARKIN-independent mitophagy by regulating AMBRA1 activation *via* IKKα

Di Rita et al.

Supplementary Figure 1

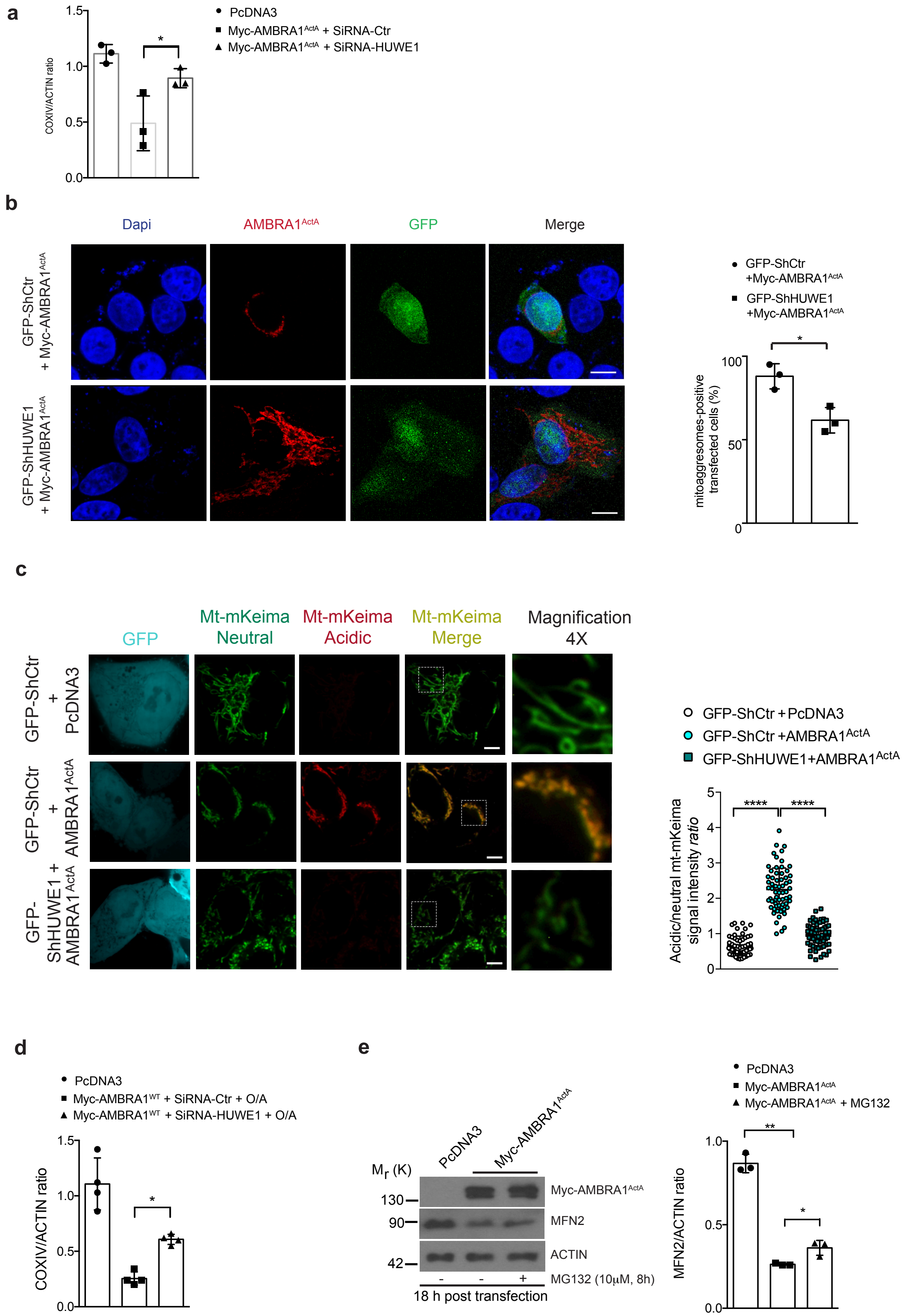

Supplementary Figure 1: The inhibition of HUWE1 E3 ligase impairs AMBRA1-mediated mitophagy

a. The graph shows the COXIV/ACTIN *ratio* in AMBRA1^{ActA}-transfected cells, depleted of HUWE1 (SiRNA-HUWE1). n = 3. **b**. HeLa cells co-transfected with vectors encoding for Myc-AMBRA1^{ActA}+GFP-ShCtr or Myc-AMBRA1^{ActA}+GFP-ShHUWE1 were immunostained for Myc (red) and GFP (green) antibodies by confocal microscopy. n = 3. SiRNA-Ctr = 9 individual fields; SiRNA-HUWE1 = 9 individual fields. Scale bar 10 µm. **c**. mt-mKeima PINK1-KO cells were transfected with the empty vector PcDNA3 or a vector coding Myc-AMBRA1^{ActA} in combination with GFP-ShCtr or GFP-ShHUWE1. Live cells were then analysed using time-lapse imaging. The GFP was pseudo-coloured in cyan in order to detect the acidic-mt-mKeima (as red signal) and neutral-mt-mKeima (as green signal). The graph shows the acidic/neutral mt-mkeima signal intensity evaluated in GFP-ShCtr+PcDNA3 (white spheres), or GFP-ShCtr+Myc-AMBRA1^{ActA} (cyan spheres), or GFP-ShHUWE1+Myc-AMBRA1^{ActA} (green squares). Each point represents the mean of GFP-positive cells mt-mKeima signal in a single field. n = 3. Scale bar 10 µm. **d**. The graph shows the COXIV/ACTIN *ratio* in AMBRA1^{WT}-transfected cells, expressing a SiRNA-HUWE1. n = 3. **e**. HeLa cells transfected with Myc-AMBRA1^{ActA} in combination or not with HUWE1 vector (18 hrs) and treated with MG132 (10 µM, 8 hrs) were subjected to western blot analysis, looking to MFN2 levels. n = 3. The quantification results as the mean of experimental triplicate (± S.D). *P<0.05; **P<0.01; ****P<0.0001. Statistical analysis was performed using One-Way ANOVA (**a**, **c**, **d, e**) or Student's t-test (**b**). M_r(K) = relative molecular mass expressed in Kilo Dalton.

Supplementary Figure 2

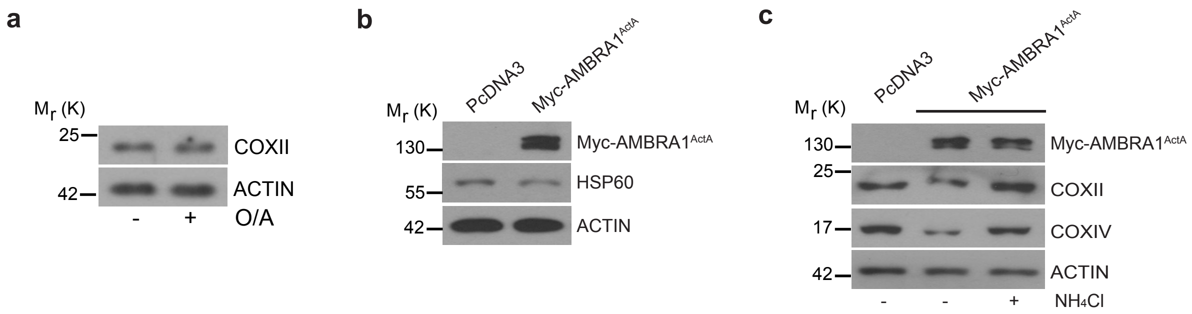

Supplementary Figure 2: AMBRA1 induces mitophagy in Penta-KO cells

a. Penta-KO cells were treated with O/A (10 µM/4 µM, supplemented with 20 µM QVD). **b.** Penta-KO cells were transfected with Myc-AMBRA1^{ActA} for 24 hrs. Protein levels of the matrix mitochondrial factor HSP60 were analysed by western blot. n = 2. **c.** Myc-AMBRA1^{ActA} Penta-KO transfected cells were treated or not with NH_4Cl after six hours of transfection. COXII and COXIV protein levels were analysed by western blot. n = 1. Mr(K) = relative molecular mass expressed in Kilo Dalton.

Supplementary Figure 3

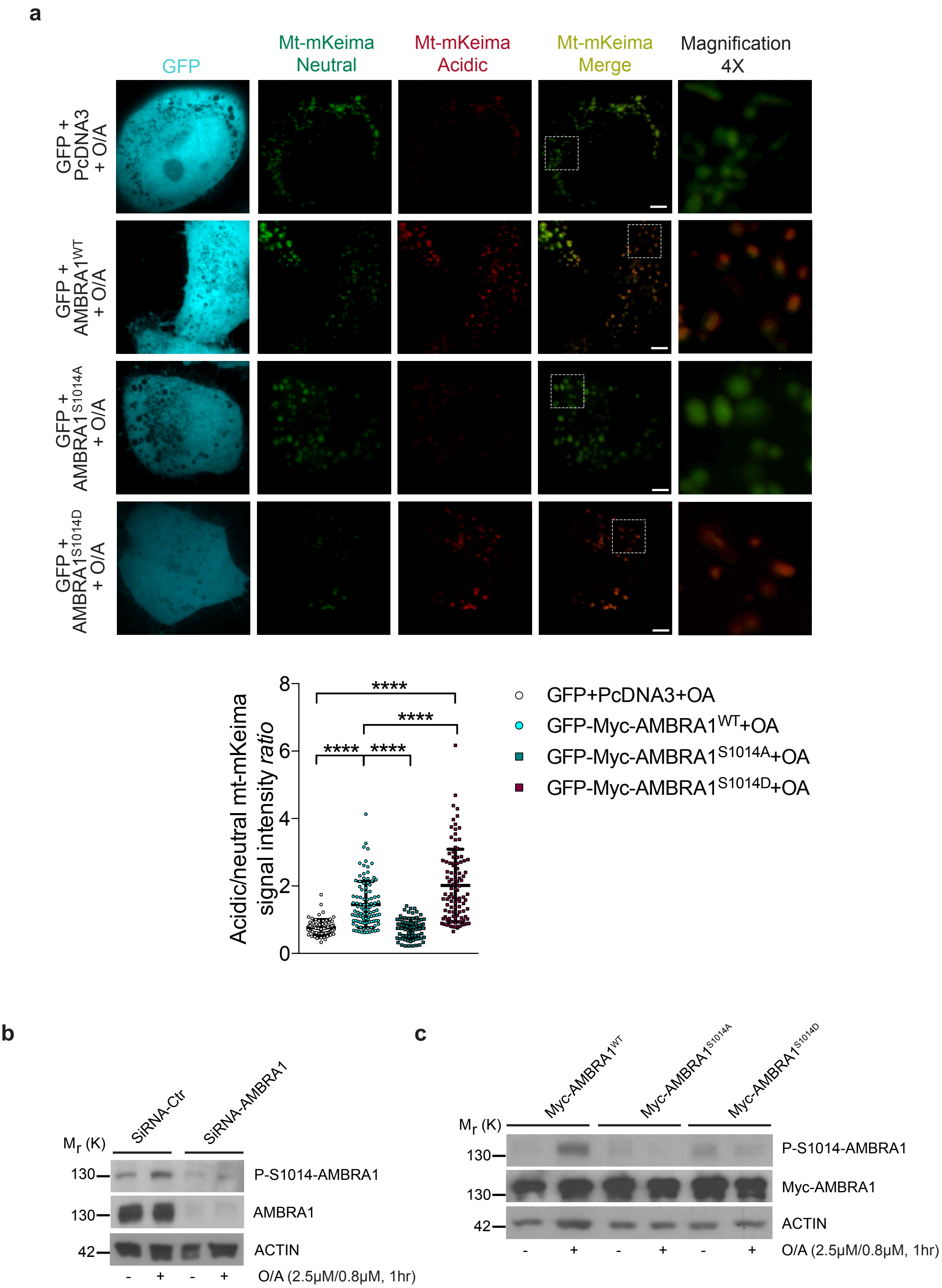

Supplementary Figure 3: The AMBRA1 phosphorylation at S1014 during mitophagy

a. mt-mKeima HeLa cells were transfected with a vector coding for GFP in combination with PcDNA3, or Myc-AMBRA1^{WT}, or Myc-AMBRA1^{S1014A}, or Myc-AMBRA1^{S1014D}. Following O/A treatment, live cells were then analysed using time-lapse imaging. The GFP was pseudo-coloured in cyan in order to detect the acidic-mt-mkeima (as red signal) and neutral-mt-mkeima (as green signal). The graph shows the acidic/neutral mt-mkeima signal intensity evaluated in GFP+PcDNA3 (white spheres), or GFP+Myc-AMBRA1^{WT} (cyan spheres), or GFP+Myc-AMBRA1^{S1014A} (green squares), or GFP+Myc-AMBRA1^{S1014D} (red squares). Each point represents the mean of GFP-positive cells mt-mkeima signal in a single field. n = 3. Scale bar 10 µm. The quantification results as the mean of experimental triplicate (± S.D). ****P<0.0001. Statistical analysis was performed using One-Way ANOVA. **b**. HeLa cells were transfected with SiRNA-Ctr or SiRNA-AMBRA1. After 24 hrs, cells were treated with FCCP (10 µM, 1 hr). By western blot analysis, we tested AMBRA1 phosphorylation in these samples. n = 1. **c**. HeLa cells were transfected with vectors encoding for Myc-AMBRA1^{WT}, Myc-AMBRA1^{S1014A} or Myc-AMBRA1^{S1014D}. After 24 h, cells were treated with FCCP (10 µM, 1 hr) and we analysed AMBRA1 phosphorylation on S1014 by western blotting. n = 1. M_r(K) = relative molecular mass expressed in Kilo Dalton.

Supplementary Figure 4

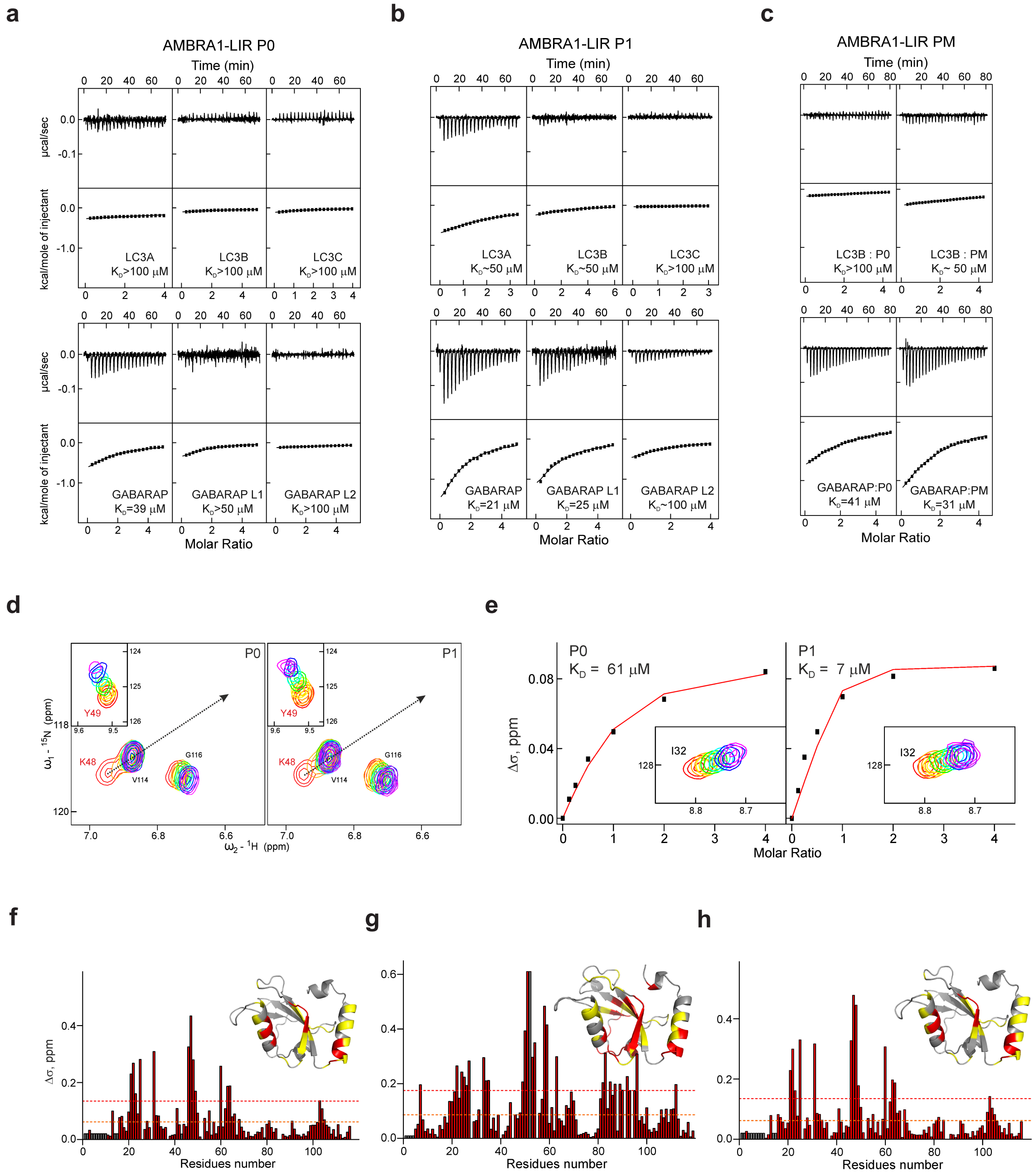

Supplementary Figure 4: AMBRA1-S1014 phosphorylation enhances the AMBRA1-mATG8s binding

a. ITC titrations of non-modified AMBRA1-LIR peptide (P0) into LC3- and GABARAP-subfamily proteins (top and bottom panels). **b.** ITC titrations of S1014-phosphorylated AMBRA1-LIR peptide (P1) into LC3- and GABARAP-subfamily proteins (top and bottom panels). **c.** Interaction of LC3B and GABARAP (upper and lower plots) proteins with P0 and with S1014D AMBRA1-LIR (PM) peptides. All measurements were performed at 25°C. The top diagrams display the raw measurements and the bottom diagrams show the integrated heat per titration step. Best fit is shown as a solid line and the K_D values are indicated. **d.** Representative sections of HSQC spectra for ^{15}N-labeled GABARAP upon titration with P0 and P1 AMBRA-LIR peptides. Both plots show fingerprint regions of the GABARAP spectra (around HN resonance of GABARAP K48-K51 in LC3B; neighbouring GABARAP residue Y49 is shown in a box). Molar ratios of protein:peptide are rainbow colour coded (1:0, 1:0.125, 1:0.25, 1:0.5, 1:1, 1:2 and 1:4; from red to magenta) for each titration step. CSP for K51 and V58 HN backbone resonances are stressed with the arrows. **e.** K_D values calculated for the GABARAP residue I32 upon titration with P0 and P1 (left and right plots) AMBRA-LIR peptides. Original CSP values are shown as black squares, resulting fit is given as a red line in each plot. The original HSQC areas around I32 HN resonance are shown as a box under fitted curves. **f.** CSP values (*Δδ*) at the last titration stages for GABARAP protein with P0 peptide are plotted against residues numbers. The orange dashed lines indicate the standard deviations (σ) over all residues within each dataset, the red dashed lines indicate double σ values, and grey bars represent residues with non-assigned HN resonances. The CSP values mapped on the GABARAP protein structure (ribbon diagrams, PDB ID 1GNU) are shown in the upper right corner.

Residues with small (*Δδ*<*σ*), intermediate (*σ*<*Δδ*<2*σ*) or strong (2*σ*<*Δδ*) CSPs were marked in grey, yellow and red, respectively. **g.**, **h.** Mapping of *Δδ* values for LC3B (**g**) and GABARAP (**h**) titrations with P1 AMBRA1-LIR on protein sequences and structures.

Supplementary Figure 5

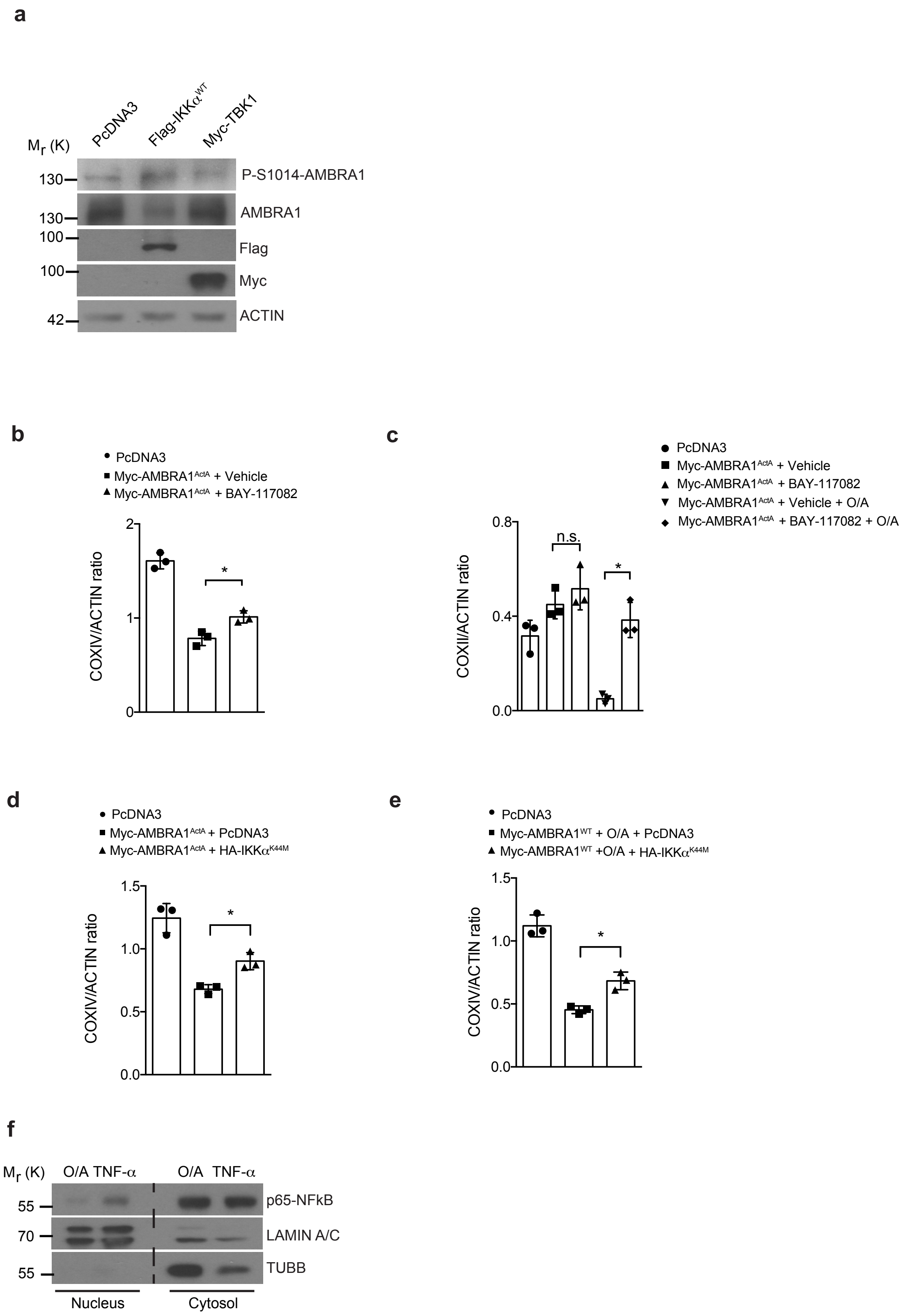

Supplementary Figure 5: IKKα role in AMBRA1-mediated mitophagy

a. HeLa cells transfected with vectors encoding for Flag-IKKα or Myc-TBK1 were treated with O/A for 1 hr and blotted for the indicated antibodies. n = 1. **b**., **c**., **d**., **e**. n = 3. **f**. Nuclear-Cytosol translocation of p65 in O/A or TNF-α-treated cells, analysed by western blot. n = 2. **f**. All graphs represent the mean of three independent experiments (± S.D.). *P<0.05. Statistical analysis was performed using One-Way ANOVA (**b**, **c**, **d, e**). M_r(K) = relative molecular mass expressed in Kilo Dalton.

Fig. 1b

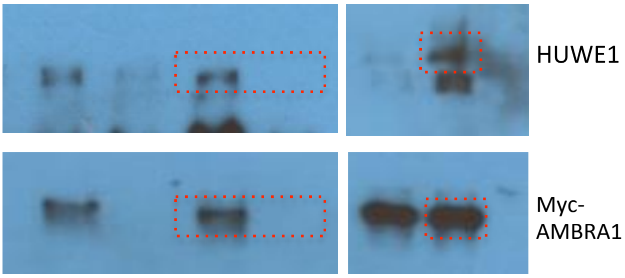

Fig. 1c

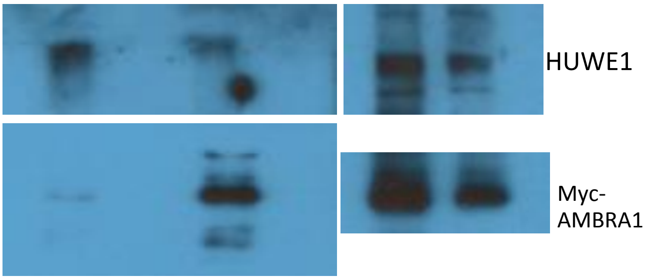

Fig. 1d

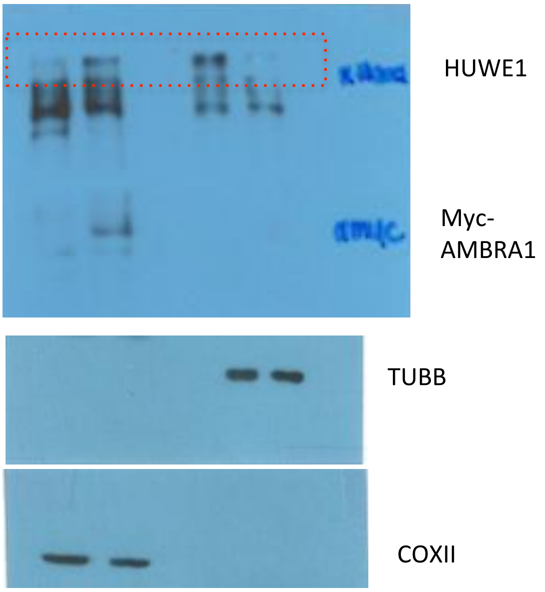

Fig. 1e

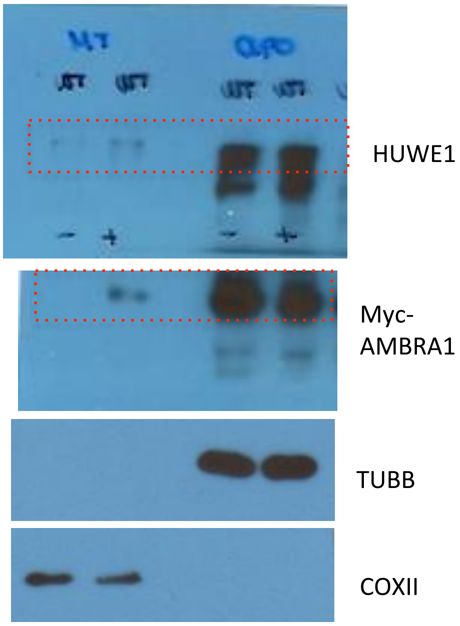

Fig. 1f

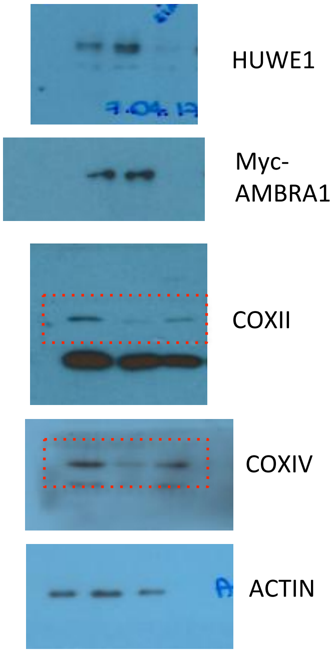

Fig. 1g

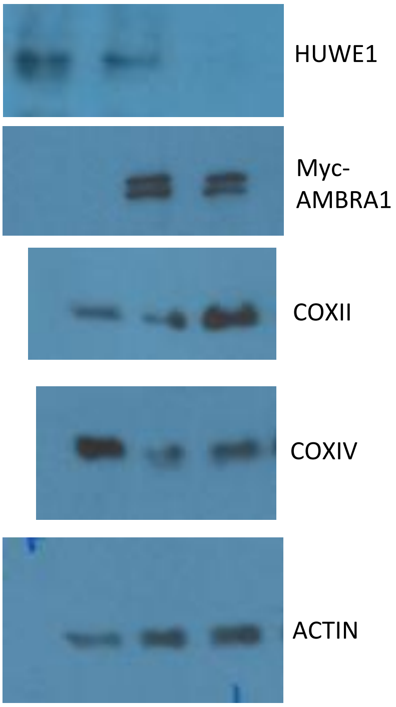

Fig. 1h

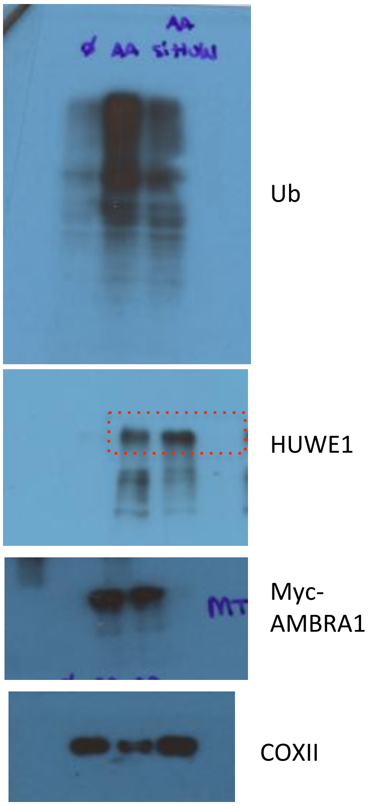

Fig. 1k

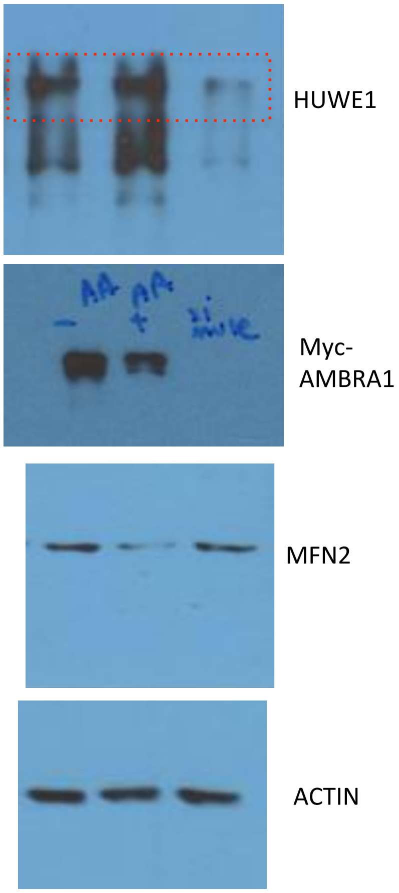

Fig. 1i

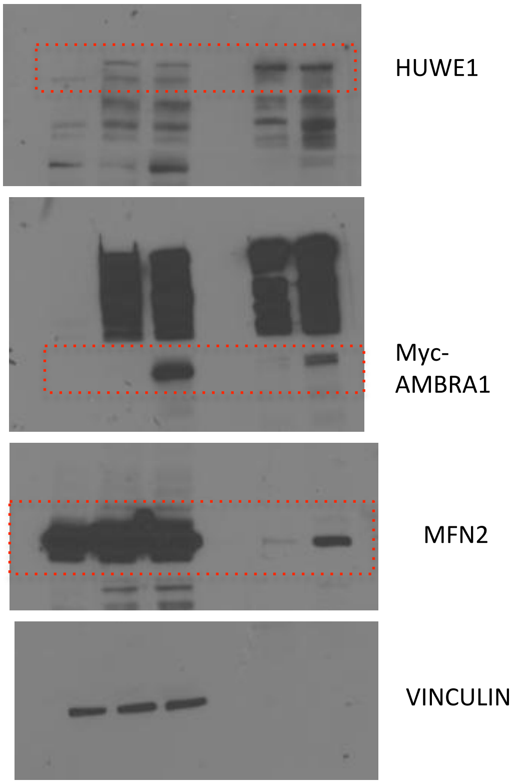

Fig. 1j

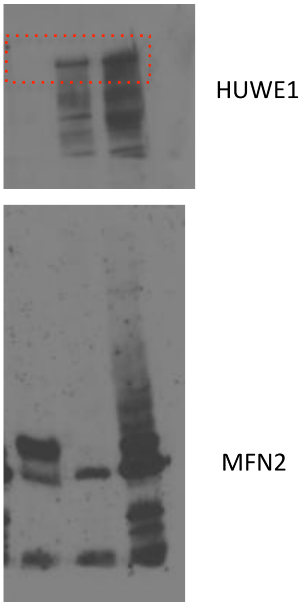

Fig. 2a

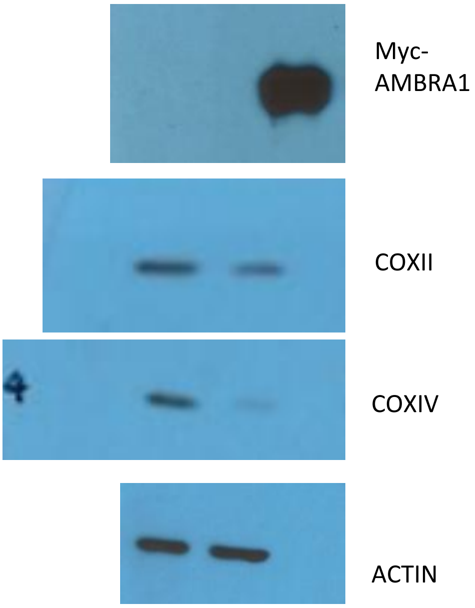

Fig. 2c

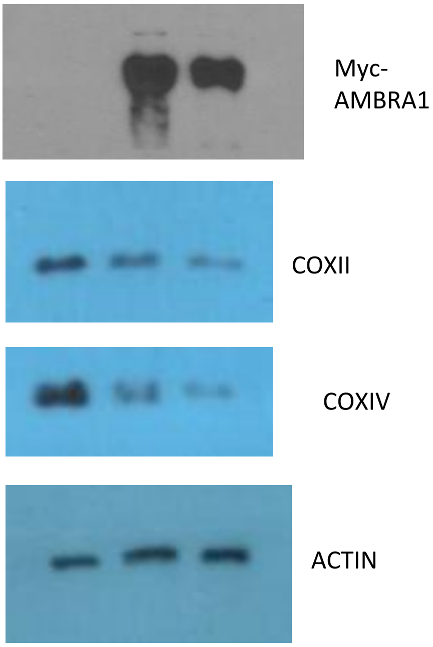

Fig. 3b

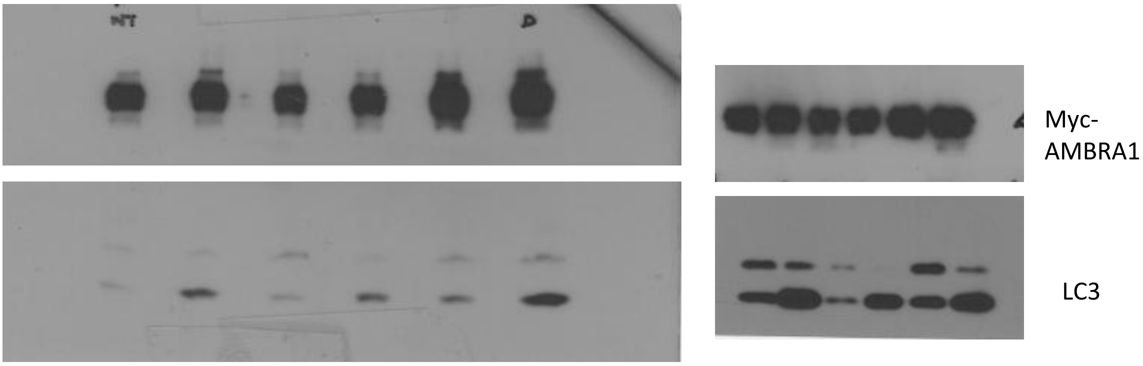

Fig. 3c

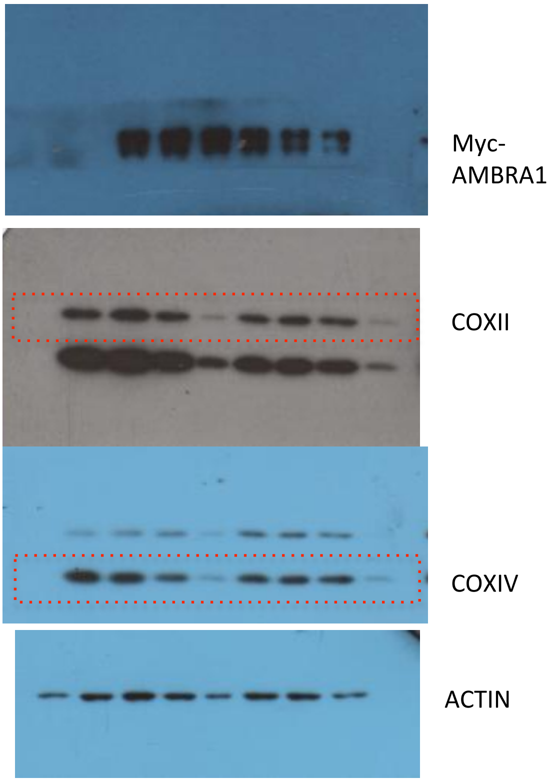

Fig. 3e

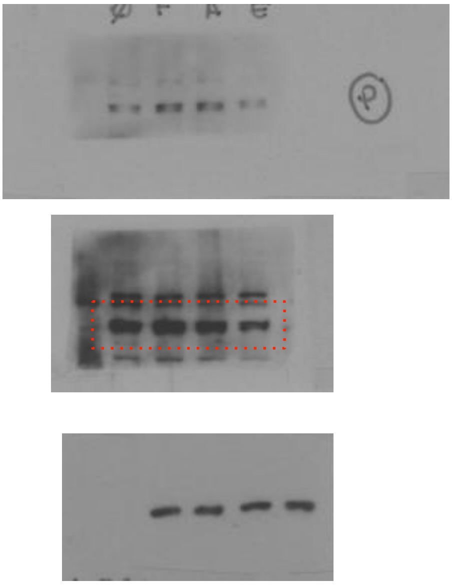

Fig. 5b

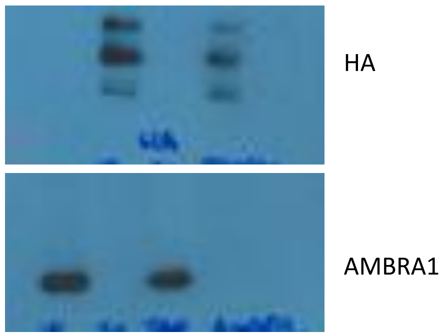

Fig. 5c

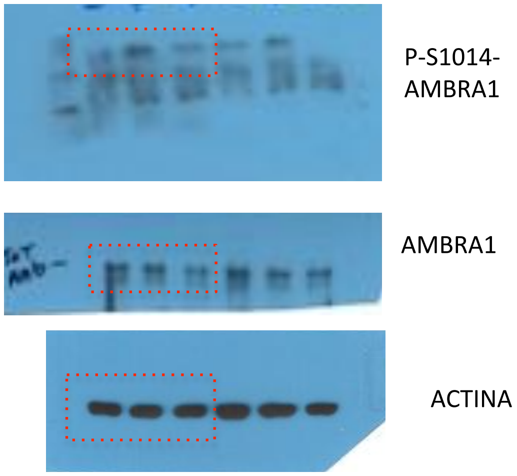

Fig. 5d

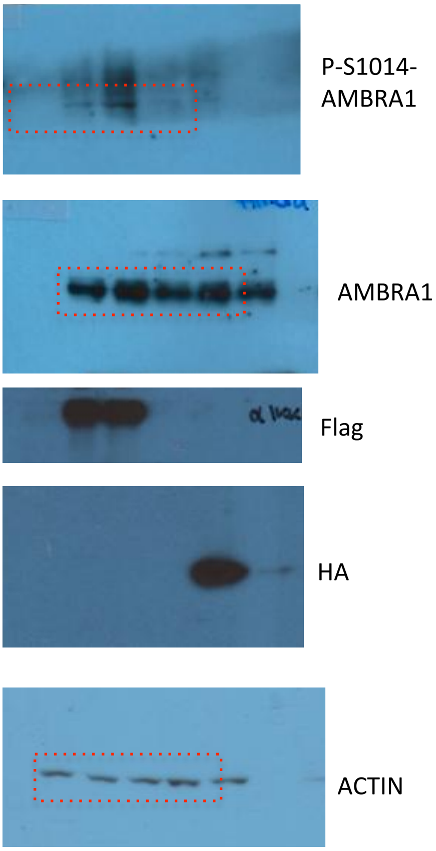

Fig. 5e

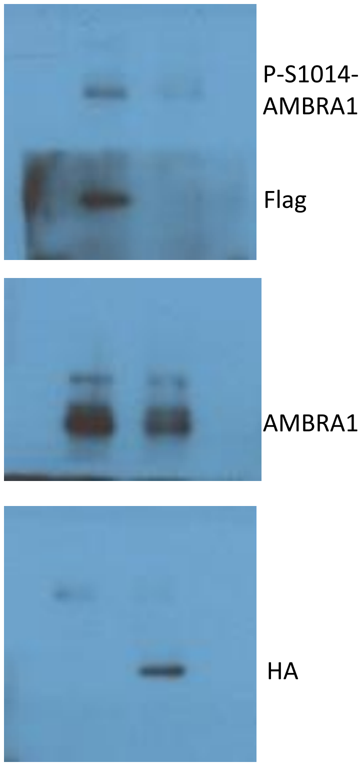

Fig. 5f

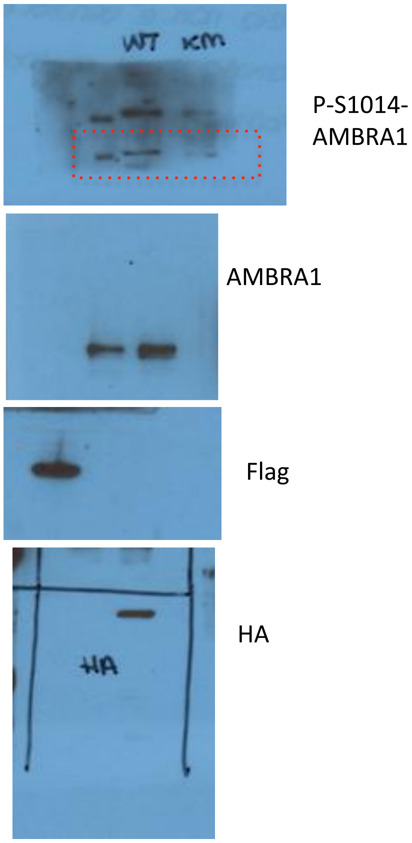

Fig.5g

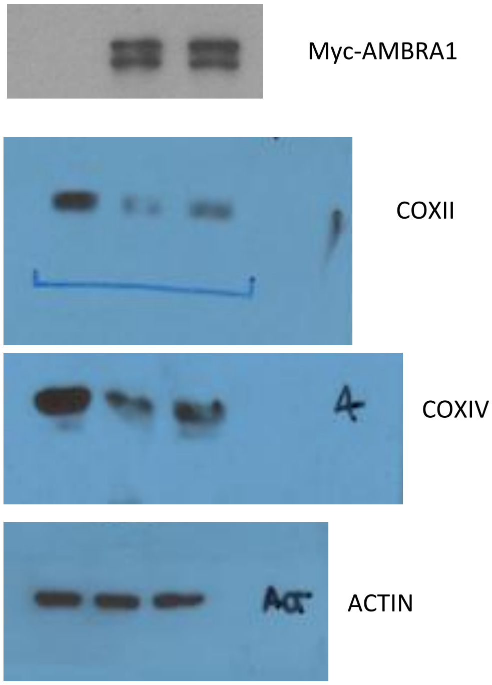

Fig.5h

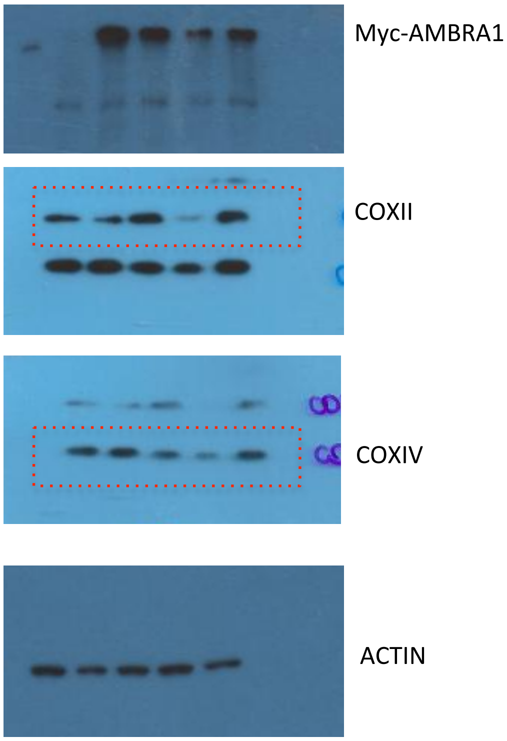

Fig.5i

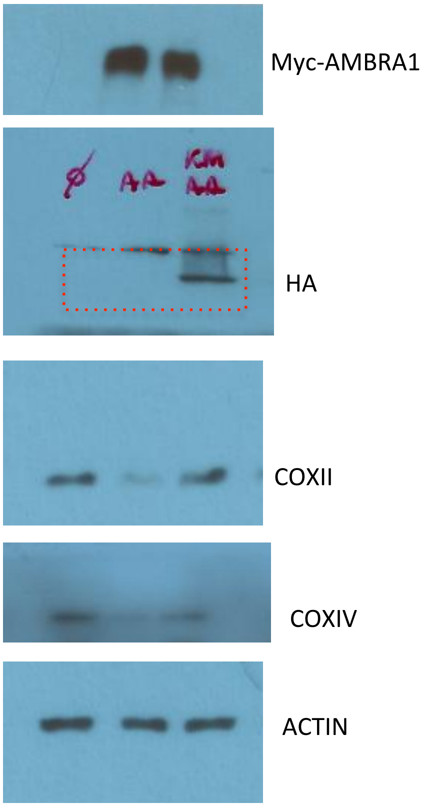

Fig.5j

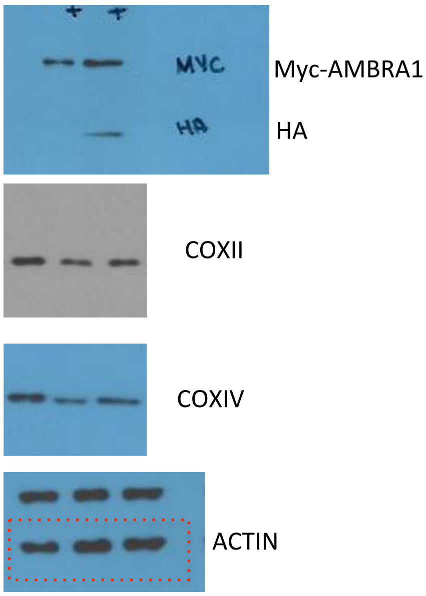

Fig.6a

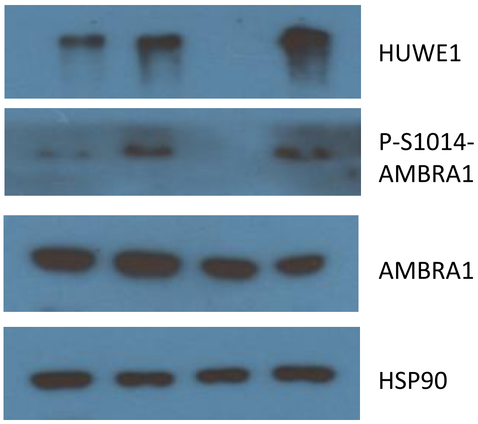
HUWE1
P-S1014-
AMBRA1
AMBRA1
HSP90

Fig.6b

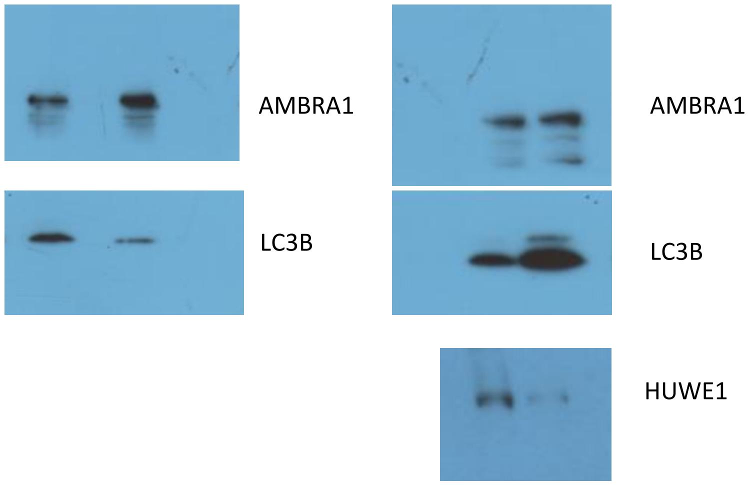
AMBRA1
LC3B
AMBRA1
LC3B
HUWE1

Fig.7a

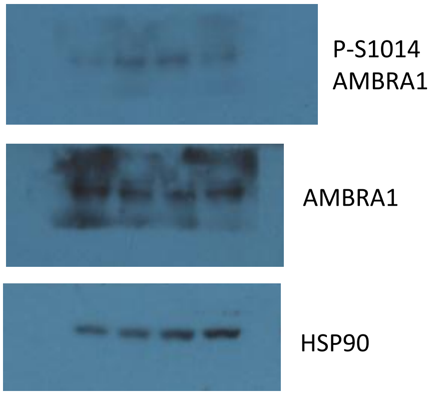

Fig.7b

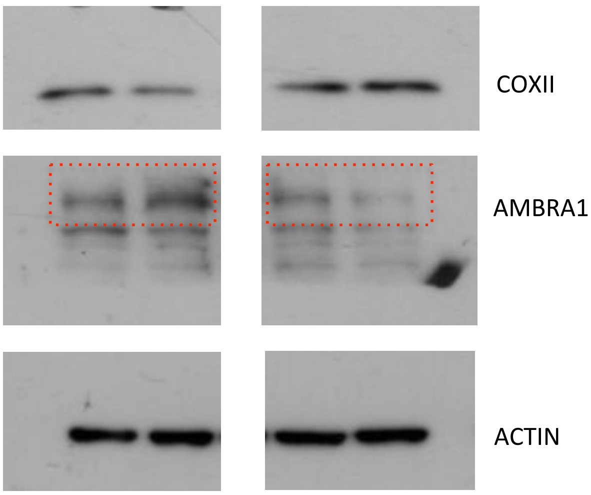

Fig.7c

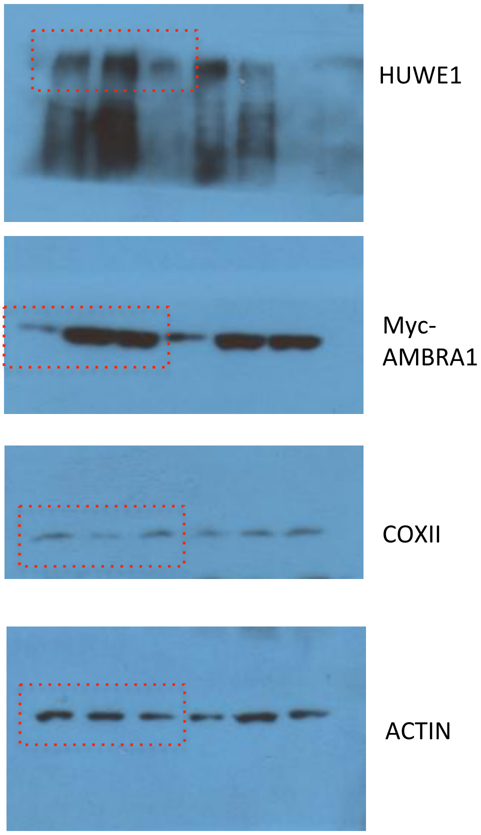

Fig.7d

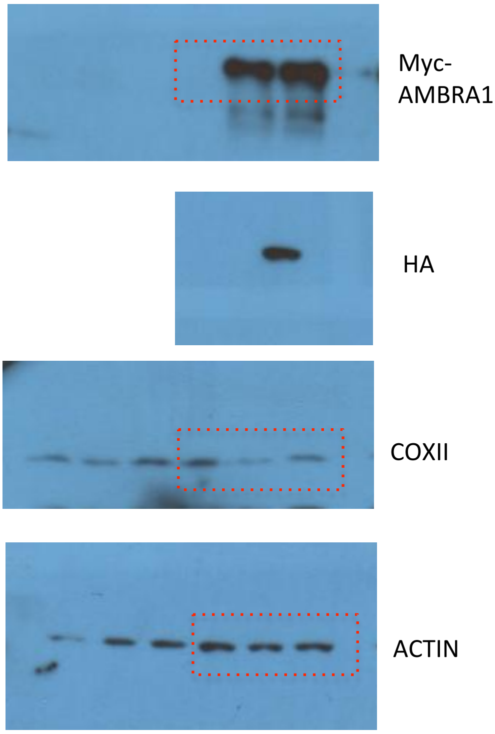

Supplementary Fig.1e

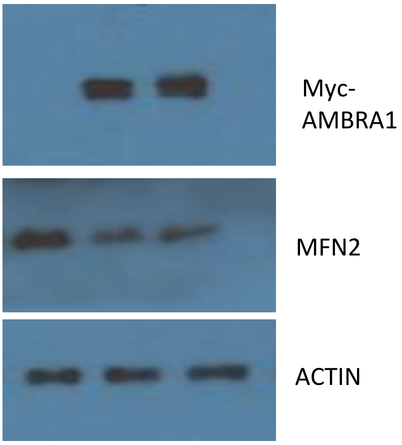

Supplementary Fig.2a

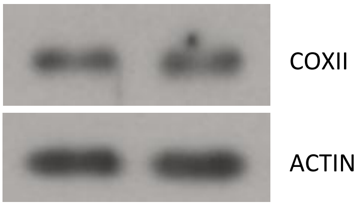

Supplementary Fig.2b

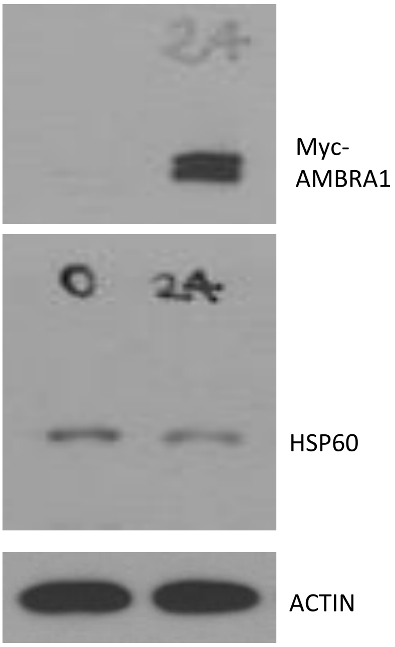

Supplementary Fig.2c

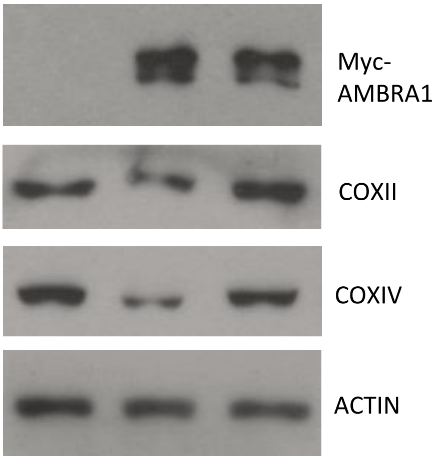

Supplementary Fig.3b

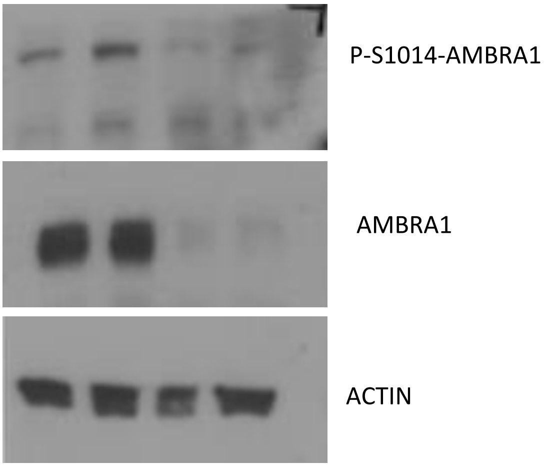

Supplementary Fig.3c

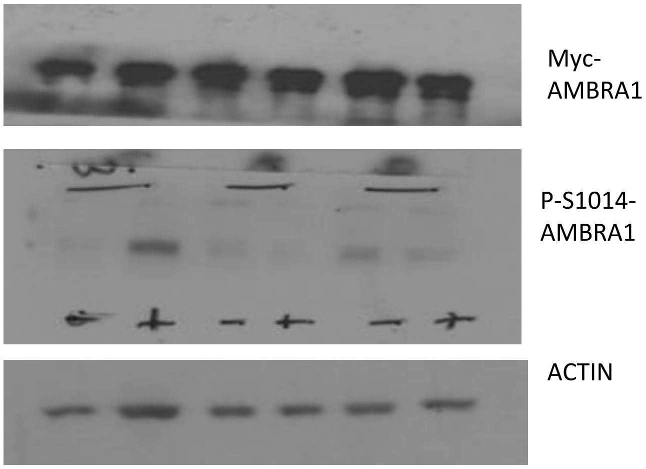

Supplementary Fig.5a

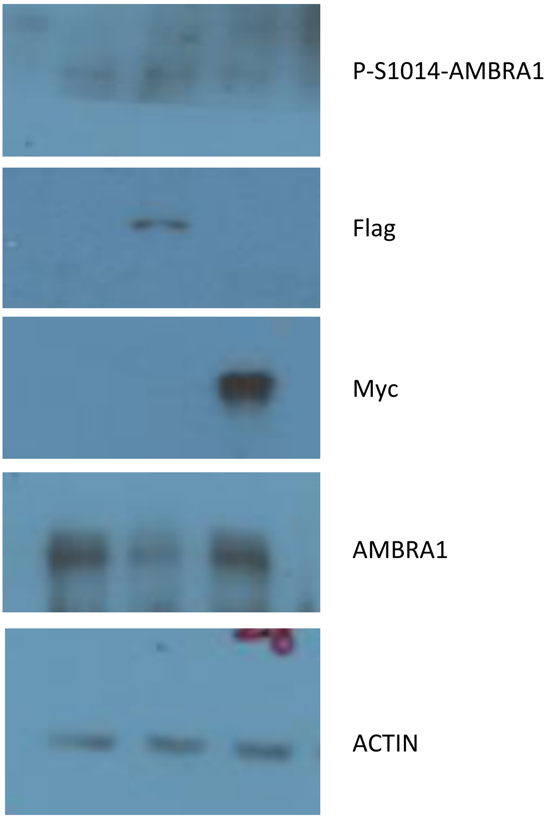

Supplementary Fig.5f

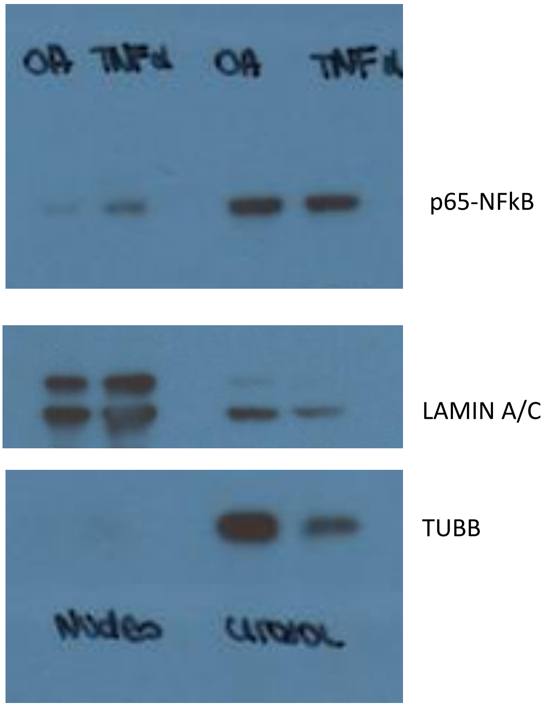

Supplementary Figure 6. Uncropped scans of the Western blots shown in the indicated figures.

Table 1. List of antibodies used for western blot (WB) and immunofluorescence (IF) analysis

Antibody	Company	Catalogue Number	Species	Dilution WB	Dilution IF
ACTB/beta actin	Sigma-Aldrich	A2228	rabbit	1:2000	
AMBRA1	Novus	26190002	rabbit	1:1000	
AMBRA1	Santa Cruz Biotechnology	sc-398204	mouse	1:1000	
COXII	Abcam	ab110258	mouse	1:2000	
COXIV	Abcam	ab33985	mouse	1:5000	
Flag	Sigma-Aldrich	F7425	rabbit	1:1000	
Flag	Sigma-Aldrich	F3165	mouse	1:3000	
GFP	Santa Cruz Biotechnology	sc-8334			1:100
HA	Sigma-Aldrich	H3663	mouse	1:1000	
HA	Sigma-Aldrich	H6908	rabbit	1:1000	
HSP60	Santa Cruz Biotechnology	sc-13966	rabbit	1:2000	
HSP90	Santa Cruz Biotechnology	sc-7947	rabbit	1:2000	
HUWE1	Bethyl	A300-486A	rabbit	1:2000	
LAMIN A/C	Santa Cruz Biotechnology	sc-20681	rabbit	1:2000	
LC3B	Cell Signaling	#2775	rabbit	1:1000	
Myc	Santa Cruz Biotechnology	sc-40	mouse	1:1000	1:100
P-S1014-AMBRA1	Covalab		rabbit	1:1000	
p65 (NF-kB)	Cell Signaling	#8242	rabbit	1:1000	
TOM20	Santa Cruz Biotechnology	sc-FL145	rabbit	1:1000	1:300
TUBB	Sigma-Aldrich	T6199	mouse	1:5000	
Ub	Santa Cruz Biotechnology	sc-8017	mouse	1:1000	
VINCULIN	Sigma-Aldrich	V9131	mouse	1:1000	

Table 2. List of plasmids and mutagenesis products

Plasmid	Mutation	Description
PcDNA3	None	Empty vector
Mito-DsRED	None	PcDNA3 vector containing human Cox8A mitochondria signal fused with wild type DsRED protein
Myc-AMBRA1^{WT}	None	Myc-tagged AMBRA1 sequence codifying for the wild type form of AMBRA1
Myc-AMBRA1^{S1014A}	S1014A	Myc-tagged AMBRA1 sequence codifying for the phospho-dead form of AMBRA1
Myc-AMBRA1^{S1014D}	S1014D	Myc-tagged AMBRA1 sequence codifying for the phospho-mimetic form of AMBRA1
Myc-AMBRA1^{ActA}	ActA sequence addicted to AMBRA1	Myc-tagged AMBRA1 sequence is fused with ActA sequence (as described in Strappazzon et al., 2015)
Flag-IKKα	None	Flag-tagged IKKα sequence codifying for the wild tyoe form of IKKα
HA-IKKα^{K44M}	K44M	HA-tagged Kinase dead form of IKKα
TBK1-Myc	None	Myc-tagged TBK1 kinase
pENTR1A-HUWE1	None	Untagged HUWE1 wild type construct<br>was a gift from Jean Cook (Addgene plasmid # 37431)
GFP-ShHUWE1	None	HUWE1 construct in lentiviral GFP vector
GFP-ShCtr	None	Scrambled shRNA cassette in pGFP-C-shLenti Vector

Table 3. List of primers sequences for point mutations

Construct	Primer Sequences	Type of mutation
Myc-AMBRA1^{S1014A}	5'-CGACCAGAGGCCTTAAACGCTGGTGTTGAGTACTACTGGGAC-3'<br>5'-GTCCCAGTAGTACTCAACACCAGCGTTTAAGGCCTCTGGTCG-3'	Point mutation S→A
Myc-AMBRA1^{S1014D}	5'-CGACCAGAGGCCTTAAACGATGGTGTTGAGTACTACTGGGAC-3'<br>5'-GTCCCAGTAGTACTCAACACCATCGTTTAAGGCCTCTGGTCG-3'	Point mutation S→D

Table 4. List of oligos RNA interference

RNA interference	Sequence	Source
SiRNA-AMBRA1	5’-GGCCUAUGGUACUAACAAAUU-3’<br>5’-UUUGUUAGUACCAUAGGCCUU-3’	ThermoFisher Lifescience
SiRNA-HUWE1	5’-GCAGAUAAAUCUGAUCCUAAACCTG-3’<br>3’-UUCGUCUAUUUAGACUAGGAUUUGGAC-5’	Integrated DNA Technologies (IDT) #150971213
SiRNA-HUWE1	5’-AAGCCCUUCUGAAAUCAUGGAAUCT-3’<br>3’-CUUUCGGGAAGACUUUAGUACCUUAGA-5’	Integrated DNA Technologies (IDT) #150971216

Supplementary Note 1

ITC experiments show that the non-modified AMBRA1-LIR (P0) binds to all 6 mATG8 proteins with low affinity (Supplementary Fig, 4a, upper plots), exhibiting some preference to GABARAP-subfamily proteins. Strongest interaction appears to the GABARAP protein, with K_D of ~40 mM, while for the LC3- proteins and for GABARAPL2 K_D values can be only estimated. The binding enthalpy for all mATG8 proteins is small, defining the entropy as a main driving force of the interactions. However, both phosphorylation of S1014 (P1, Supplementary Fig. 4b) either introduction of phospho-mimicking aspartate to this position (PM, Supplementary Fig. 4c) increase affinity of AMBRA1-LIR interaction with mATG8 analogues (K_D values decreases ~2-5 times). For the GABARAP protein K_D shifts down to 21 mM as determined by ITC. Also ITC titration of P1 AMBRA1-LIR peptide to LC3B, which is characterized by very low enthalpy and is almost invisible at 25°C (Supplementary Fig. 4a and 4b, LC3B plots), shows unambiguously increase of LC3B affinity to AMBRA1-LIR upon S1014 phosphorylation when performed at 35° (Fig. 4a, K_D decrease from >100 to 53 mM).

In order to characterize the AMBRA1-LIR interaction with LC3- and GABARAP-family proteins in more details, we performed NMR titration experiments, in which the AMBRA1-LIR peptides of different phosphorylation states (P0 and P1) were stepwise added to ^{15}N-labelled LC3B and GABARAP proteins (Fig. 4b-4d and Supplementary Fig. 4d-h). We observed fast exchange behaviour of backbone HN resonances for almost all residues in LC3B and GABARAP proteins upon titrations (in agreement with the high entropy contribution from our ITC data); however, the P1 induces perturbations of the key LC3B/GABARAP resonances with fast-to-intermediate exchange regime in contrast to P0

(fast exchange regime, Fig. 4b-4c and Supplementary Fig. 4d-4e). K_D values, calculated from chemical shift perturbations (CSP), for LC3B and GABARAP interactions with P0 and P1 peptides, mirror that from ITC experiments (providing more trustable K_D of ~150 mM for LC3B:P0 interaction). We also mapped the CSP observed upon P0 and P1 titrations on the sequence and structure of the LC3B and GABARAP proteins (Fig. 4d and Supplementary Fig. 4f-4h). Comparison of the CSP induced by P0 and P1 titrations (for both LC3B and GABARAP proteins) indicates no significant differences in residues affected or directions of perturbations. It means that the increased affinity of AMBRA1-LIR peptide to the mATG8 proteins upon phosphorylation of S1014 residue is mediated not by specific conformational changes of mATG8 proteins backbone, rather by electrostatic interactions and salt bridges between phosphoryl group and side-chains of corresponding proteins (similar to OPTN-LIR phosphorylation,[23]). This conclusion is further supported in our MD simulation experiments (Fig. 4f).
